# Hemorrhagic shock after dental extractions in a patient with anticoagulant and antiplatelet therapy: a case report

**DOI:** 10.1016/j.ijscr.2025.111375

**Published:** 2025-04-25

**Authors:** Alexia Pouplin, Diane Pham Kormann, Gilles Dolivet, Bérengère Phulpin

**Affiliations:** a Université de Lorraine, CHRU-Nancy, Service d'Odontologie, Département de chirurgie orale, F-54000 Nancy, France; b Université de Lorraine, Faculté d'Odontologie, F-54500 Nancy, France; c Institut de Cancérologie de Lorraine, Unité de chirurgie cervico-faciale et dentaire, F-54519 Vandoeuvre-lès-Nancy, France; d Université de Lorraine, CNRS, CRAN, F-54000 Nancy, France

**Keywords:** Tooth extraction, Hemorrhagic shock, Antiplatelet agent, Heparin, Major bleeding

## Abstract

**Introduction:**

Hemorrhagic shock requiring red blood cell transfusion following tooth extraction is rare.

**Case presentation:**

We report the case of a 71-year-old patient who was treated at the hospital for hemorrhagic shock the day after the extraction of two teeth and for a second episode seven days after the surgery. The patient was treated in oncology and his medication included acetylsalicylate of DL-Lysine and Tinzaparine. The pre-operative biological assessment that allowed the surgery and validation of the action to be taken was provided by the cardiologist. Treatment consisted in transfusion and wound revision with controlled hemostasis.

**Discussion:**

Alveolar bleeding leading to two episodes of hemorrhagic shock (on day 1 and day 7) is an exceptionally rare occurrence. Even if the patient had some risk factors increasing the risks of bleeding, including age, anticoagulants, oncological treatments and recent teeth extractions, no case in the scientific literature was found with similar conditions and no explanation justifying the intensity of the hemorrhage.

**Conclusion:**

Precautions for hemostasis during the intervention are important but sometimes insufficient. For patients at high risk of bleeding with comorbidity like anticoagulant and antiplatelet therapy, it is necessary to establish 7-day postoperative monitoring to control possible post-operative bleeding.

## Highlights

• Hemorrhagic shock following tooth extraction is rare.
• The pre-operative biological assessment allowed the surgery and the course of action was validated by the oncologist.
• Even if the patient had risk factors for bleeding, no explanation justifying the intensity of the hemorrhage.
• There are no guidelines in oral surgery to manage patients receiving oral anticoagulants combined with antiplatelet therapy.

## 1 Introduction

Hemorrhagic shock after dental invasive procedure is a rare occurrence and is characterized by a significant and rapid extravascular loss of blood, that leads to a decrease in circulating blood volume. Clinically, an hemorrhagic shock is defined as an acute and lasting decrease in systolic blood pressure (SBP) with values below 90 mmHg (or 30 % below the patient’s usual SBP values). It is accompanied by clinical signs of hypoperfusion, organ dysfunctions or failures.

Tooth extractions in one sector are considered a low risk of hemorrhagic surgery [1] and, in most cases, major bleeding can be controlled with local hemostatic measures without blood transfusion during the intervention [[2], [3], [4]].

Patients with anticoagulant or antiplatelet therapy have a higher risk of bleeding in oral surgery, and guidelines exist to help surgeons for treatment management. Thus, for patients with antiplatelet therapy, the SFCO (Société française de chirurgie orale) does not recommend stopping the treatment for monotherapy. Regarding curative heparin anticoagulant, the recommendation for low risk hemorrhagic surgery is to maintain the treatment if the patient receives an injection per day [1]. These recommendations have some limitations in case of concomitant anticoagulant and antiplatelet therapy.

This case describes two hemorrhage shock (on day 1 and day 7) after dental extraction performed in a patient with acetylsalicylate of DL-Lysine (Kardegic®) and tinzaparine (Innohep®) sodique medication. This case has been reported in line with SCARE criteria [5].

## 2 Case presentation

A 71 years old man consulted for an oral dental assessment before a Denosumab (XGEVA®) treatment. He suffered from prostate carcinoma metastatic at the lymph node, bone level treated with chemotherapy by docetaxel associated with androgen suppression. He also presented an ischemic heart disease requiring angioplasties and stent. Because of atrial fibrillation with major embolic risk, he was treated with anticoagulant. His medical treatments the day of surgery included acetylsalicylate of DL-Lysine (75 mg) and Tinzaparine 14,000 U per day. In consultation with the prescribing doctor, the oral surgery was possible in the hospital context without interrupting his medications.

Anticoagulant treatments were modified 2 months before surgery. Indeed, Tinzaparine was introduced two months before the operation by the cardiologist due to a permanent and persistent atrial fibrillation, with major embolic risk that needed a curative anticoagulant therapy, in addition to his usual antiplatelet treatments: acetylsalicylate of DL-Lysine 75 mg per day and clopidogrel 75 mg per day. Another modification of the medication was realised one month before the operation. Indeed, clopidogrel was stopped following episodes of epistaxis that resolved spontaneously. The control biological assessment showed grade II anemia (corresponding to moderate anemia: hemoglobin 8.0–10.9 g/dL according to WHO Classification of Anemia Severity) so a transfusion of 2 units of red blood cell was performed. Twenty days before the dental extraction, no episode of epistaxis occurred after the discontinuation of the clopidogrel treatment. A control biological assessment was performed and showed grade II anemia with 8,4 g/dL. So, in this context, a new transfusion of 2 units of red blood cell was performed and chemotherapy was deferred for two weeks.

He had diabetes type 2 insulin-requiring, hypertension and dyslipidemia. The patient was a non-smoker.

**Fig. 1:**
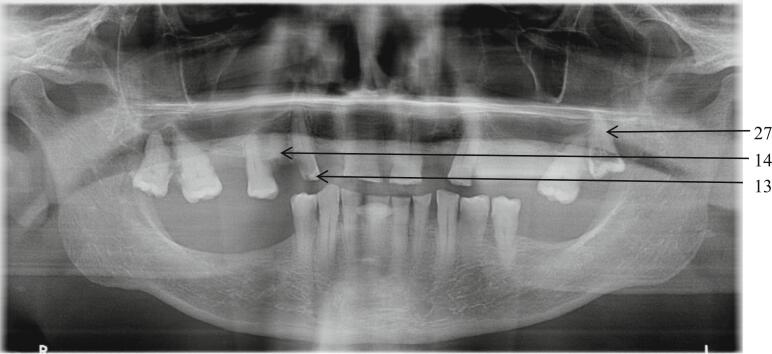
Orthopantomogram, indication for extraction of teeth n°13-14-27.

During the dental consultation, clinical examination and radiological examination (Fig. 1) were carried out and showed wide tooth decay on teeth n° 27 and n°13 that couldn't be preserved like the root of the tooth n°14. These teeth constituted infectious sites that needed to be eliminated before initiating treatment with denosumab in order to limit the risk of osteochimionecrosis.

In consultation with the oncologist, a biological assessment with cell blood count/platelet count and antibiotic prophylaxis therapy with amoxicillin 3 g/day during 6 days to start in the morning of the intervention had to be carried out before the surgical procedure

**Fig. 2:**
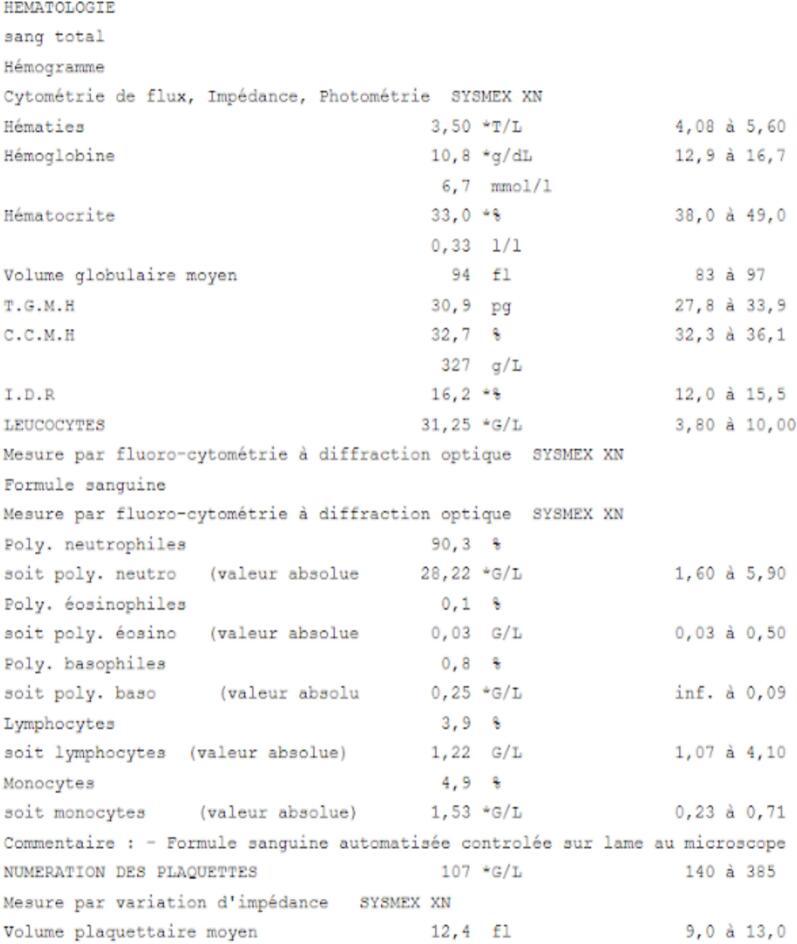
Biological assessment the day of the surgery.

The day of the operation, the patient was therefore on acetylsalicylate of DL-Lysine 75 mg and tinzaparine 14,000 U per day with the last injection subcutaneous the day before at 5p.m, so a half day before surgery as recommended by the SFCO [1]. The pre-operative biological assessment recommended by the oncologist revealed a hemoglobin of 10.8 g/dL (normal lab values 13.8–17.2 g/dL) and 107,000 platelets/μL (normal lab values 150,000–450,000 cells/μL) (Fig. 2), which was compatible with an oral surgery. The procedure was carried out under local anesthesia with a vasoconstrictor. An alveolectomy was performed next to the maxillary right canine (n°13), as well as simple avulsion for the residual root of the maxillary right first premolar (n°14). An hemostasis protocol was implemented intraoperatively with a collagen hemostatic sponge (Pangen®) inserted into the sockets and airtight sutures with simple stitches. Bleeding on the socket site of tooth n°13 was noticed, but not in the socket site of tooth n°14 and hemostasis was achieved within 5 min following the surgical procedure. Post-operative instructions were given to the patient before returning home: no spit, no mouthwash for 48 h and cold or warm food for the next 48 h.

However, the day after the operation, the patient presented to the emergency room of his usual hospital with massive bleeding localised on the alveole of tooth 13. Hemorrhagic shock was diagnosed, requiring the transfusion of 3 units of packed red blood cell, vascular filling and the use of noradrenaline. In a second stage, diuretic were used for acute pulmonary oedema. In the oral cavity, a surgery was realised by oral surgeon under local anesthesia with adrenaline. After the removal of the blood clot and a cure and a rinse, a new collagen hemostatic sponge (Pangen®) was inserted into the bleeding sockets and airtight sutures with simple stitches stopped local bleeding. The patient was subsequently transferred to geriatric ward where he presented aspiration pneumonia, treated with amoxicillin/clavulanic acid 3 g per day during 10 days and oxygen therapy 3 L/min. After 7 days of hospitalization, the patient was discharged.

In the evening of that same day, the patient was again hospitalized urgently in a context of marked hemodynamic deterioration due to blood clot loss with complications such as hypotension, significant vomiting of blood, and skin pallor. The admission examination indicated a systolic blood pressure of 67 mmHg [normal 120-129 mmHg], a heart rate around 100, psychomotor slowing, moderate polypnea and an hemoglobin of 6.2 g/dL. A second red blood cell transfusion was necessary, but was complicated by hyperthermia, which led to the interruption of the transfusion, replaced by the administration of fresh frozen plasma. Local care was carried out with the removal of clots located on the upper right gum in the extraction sites and the application of compresses soaked in Exacyl to be squeezed under the teeth. Following this new hemorrhagic episode, a new local hemostasis was performed (as described above), and a new transfusion was carried out this time without incident.

## 3 Discussion

Hemorrhagic shock requiring red blood cell transfusion following tooth avulsion is rare with only a few cases found in the scientific literature [[6], [7], [8], [9], [10]]. These cases are different from ours due to medical comorbidity that our patient doesn’t have: accidental discovery of a arterovenous malformation [7], or patients with hepatic fragility [1] (hepatitis C awaiting transplant [8], decompensated cirrhosis with hemostasis disorder [6,9]). In a different context, a case of delayed bleeding leading to hypovolemic shock in a baby operated on for lingual frenotomy has also been described [10]. In addition, our patient suffered from two hemorrhagic shocks. The second occurred seven days after surgery, most likely following the sloughing of the eschar.

The patient received only one type of chemotherapy. Docetaxel, is a chemotherapeutic agent from the taxane family. It is well known that docetaxel, it has myelosuppressive effects, including reduce blood cell production. The nadir is between days 7 and 10. Anemia and thrombocytopenia may occur in this patient. The patient had received the last dose of docetaxel 15 days before and the laboratory values from the blood test performed prior to the avulsions were deemed adequate for oral surgery. Moreover, the hepatic workup was also within normal limits. It is essential to be aware of the side effects resulting from the frequent presence of comorbid diseases particularly in the elderly [11]. However compared with use of oral anticoagulant alone, concomitant use of oral anticoagulant and anticancer drugs was not associated with an increased risk of major bleeding [12]. Nevertheless, a few cases of gastrointestinal [13] or cerebral hemorrhage have been reported in the literature [11]. However, to our knowledge, no cases of dental bleeding have been described.

Regarding denosumab, it was never introduced.

The second bleeding episode was most likely facilitated by the decline in the patient's overall condition. The hypovolemic shock, in combination with aspiration pneumonia, likely had an impact on the healing process.

On the one hand, on the surgical level, the surgical procedure was classified as a moderate hemorrhagic risk due to the extraction of 2 teeth on the same side. Indeed, extractions in several sectors increase the risk of bleeding [1,8], such as multiples extractions of more than three teeth or osteotomy [14]. During the intervention, an alveolectomy was realised on the upper right canine which can increase the bleeding [14], but there was no hemorrhagic risk in this area (distance from antral artery, and superior alveolar arteries [8]), no risk of tuberosity fracture, and bleeding was stopped at the end of the intervention.

In addition, in anticipation of bleeding, conventional surgical hemostasis with sutures, mechanical compressions, as well as the placement of absorbable local hemostats was carried out following the recommendations except for anti-fibrinolytic agent prescription applied with manual compression (tranexamic acid) [1]. This may be sufficient to manage postoperative bleeding [[2], [3], [4]]. In this case, hemostasis was achieved within 5 min following the surgical procedure only with local hemostatic sponge and sutures contrary to an hemorrhagic shock due to an arteriovenous malformation [7]. In the event of a hemorrhagic complication, wound revision with resumption of hemostasis is necessary [1]. During this surgical recovery, the hemostasis was also acquired quickly.

On the other hand, regarding the medical risk factors, we can make note of atrial fibrillation, which led to anticoagulation. Patients receiving an anticoagulant treatment are generally older than patients without treatment [14,15]. In our case, during the extractions, our 71-year-old patient was under treatment with acetylsalicylate of DL-Lysine and tinzaparine and this combined medications is considered a high hemorrhagic risk for oral surgery. Moreover, dual therapy and recurrent treatment modifications associated with side effects of chemotherapy may increase the risk of bleeding.

The biological assessment the day of the surgery allowed extraction. Indeed, hemoglobin (10,8 g/dL) (normal lab values 13.8–17.2 g/dL) was above the threshold value of 8; he showed a mild thrombocytopenia with 107,000 platelets/μL (normal lab values 150,000–450,000 cells/μL), a stable renal function and an undisturbed liver function. These results were compatible with dental surgery according to the recommendations.

The management of this patient on acetylsalicylate of DL-Lysine and tinzaparine therapy should follow the recommendations for management of patients under antithrombotic treatments. The recommendations on the perioperative management of patients on antiplatelet agent monotherapy do not require interruption of treatment in the same way as with patients on curative heparin with one injection per day [1]. In patients undergoing oral surgery, current guidelines support proceeding without anti-Xa levels, provided that the last dose is administered at an appropriate interval before the procedure. However, these recommendations don’t take into account the implementation of two concomitant anti-thrombotic treatments, but the surgery was allowed after a call to the prescribing cardiologist.

The continuation of antiplatelet therapy was not associated with a statistically significant difference in the risk of major bleeding in comparison to patients who stopped their medication [16]. The postoperative bleeding risk in dental surgery is increased for patients on oral anticoagulant therapy (RR: 2.794, 95 % CI: 1.722–4.532) [17]. If vitamin K antagonists and antiplatelet agents are combined, the results of the studies are contradictory. In some studies, the risk of bleeding in patients receiving oral anticoagulants combined with antiplatelet therapy was similar to patients receiving oral anticoagulants alone, suggesting that the incremental bleeding risk of combination therapy might not be clinically significant [18,19]. Another study suggest that the risk of major bleeding is increased in patients taking vitamin K antagonists and antiplatelet agents compared to patients taking antiplatelet agents alone (RR 1,77, 1,47-2,13) [20]. For patients on heparin, non-significant postoperative bleeding was found for patients on heparin and antiplatelet therapy and none of the patients with postoperative hemorrhage had serious bleeding requiring blood transfusion [21].

Concerning bleeding time, immediate postoperative bleeding is greater in patients on dual antiplatelet therapy rather than in patients on antiplatelet monotherapy or in patients without treatment [22], but these results are controversial [23]. And regarding delayed bleeding, they are more common with direct oral anticoagulant and vitamin K antagonists treatments than without treatment [15]. But the scientific literature is limited concerning hemorrhagic shock due to delayed bleeding under anticoagulant and antiplatelet therapy, and studies rarely compare bleeding after avulsion in patients treated with dual therapy [3,4,14,15,17]. It would be useful to have guidelines from scientific societies for the management of patients on a combination of antiplatelet agents and anticoagulants, or dual anticoagulant therapy.

To sum up, this case is unique in the intensity of the complication. Even if the patient has some factors, such as age, dual anticoagulant therapy and oncological treatments who causes hematological disorders, increasing the risk of bleeding during the extraction of two teeth, no case in the scientific literature was found with similar conditions and no explanation justifies the intensity of the hemorrhage.

## 4 Conclusion

To conclude, hémorragie shock is rare after dental intervention. Precautions for hemostasis during the intervention are important but are sometimes not enough. For high risk patients with comorbidity like anticoaguled and antiplatelet therapy, it’s necessary to establish 7-days postoperative monitoring to control potential postoperative bleeding.

## Author contribution

Conception and design of the study, or acquisition of data: G. Dolivet, B. Phulpin

Drafting the article of revising it critically for intellectual content: A. Pouplin, D. Pham Kormann, G. Dolivet, B. Phulpin

Final approval of the version to be submitted: A. Pouplin, D. Pham Kormann, G. Dolivet, B. Phulpin

## Consent

Written informed consent was obtained from the patient for publication.

## Ethical approval

Our case is exempt from ethical approval in our institution. This article has no ethical issues.

## Guarantor

Dr. Bérengère Phulpin

## Research registration number

Not necessary for this case report.

## Funding

This research did not receive any specific grant from funding agencies in the public, commercial, or not-for-profit sectors.

## Declaration of competing interest

None.
